# Preparatory Education

**Published:** 1881-07

**Authors:** 


*Preparatory Education.*—We of the Dental Schools are in no position to fling mud at our contemporaries the medical colleges. Both are sadly deficient in many respects. It is a comfort, however, to know that if, in our final examinations, we find papers with spelling suggestive of a need on the part of the writers of a term in the primary department of a grammar school, with orthography of exceedingly phonetic type, our cousins the M. D's are not all of them brilliant exceptions to this deficiency. Some of the papers lately reported to the Census Bureau contain spelling which would do honor to Webster, while again others spell "Child Bearth" as here represented, capitals and all. One addresses the Census Bureau as

"gentiel-Men," and another spells "fee maile" and "ConsuMP-tion" as here written, while "Cholory MorBis" figures with these capitals, and "Scroffular" is put down thus. One reporter writes that "some have moved away immediately after death and I have lost trace of them." One child died of "Teathing." "General infirmaty" killed one patient; "cronic Direy" another, and still another perished of "Croop and CluBfootid." Singularly most of these spell more difficult words, as cerebro-spinal meningitis, etc., correctly, probably by a reference to the dictionary.

We suppose that any of these gentlemen are eligible by virtue of their diplomas to seats in the American Medical Association, and if so that body can hardly exclude dentists on the score of ignorance.

